# In-hospital mortality among consecutive patients with ST-Elevation myocardial infarction in modern primary percutaneous intervention era ~ Insights from 15-year data of single-center hospital-based registry ~

**DOI:** 10.1371/journal.pone.0252503

**Published:** 2021-06-11

**Authors:** Kensuke Takagi, Akihito Tanaka, Naoki Yoshioka, Yasuhiro Morita, Ruka Yoshida, Yasunori Kanzaki, Naoki Watanabe, Ryota Yamauchi, Shotaro Komeyama, Hiroki Sugiyama, Kazuki Shimojo, Takuro Imaoka, Gaku Sakamoto, Takuma Ohi, Hiroki Goto, Hideki Ishii, Itsuro Morishima, Toyoaki Murohara

**Affiliations:** 1 Department of Cardiology, Ogaki Municipal Hospital, Ogaki, Japan; 2 Department of Cardiology, Nagoya University Graduate School of Medicine, Nagoya, Japan; 3 Department of Cardiology, Fujita Health University Bantane Hospital, Nagoya, Japan; Osaka University Graduate School of Medicine, JAPAN

## Abstract

**Objective:**

To clarify the association of detailed angiographic findings with in-hospital outcome after primary percutaneous coronary intervention (p-PCI) for ST-elevation myocardial infarction (STEMI) in Japan.

**Background:**

Data regarding the association of detailed angiographic findings with in-hospital outcome after STEMI are limited in the p-PCI era.

**Methods:**

Between January-2004 and December-2018, 1735 patients with STEMI (mean age, 68.5 years; female, 24.6%) who presented to the hospital in the 24-hours after symptom onset and underwent p-PCI were evaluated using the disease registries. The registry is an ongoing, retrospective, single-center hospital-based registry.

**Results:**

The 30-day mortality rate and in-hospital mortality rate were 7.7% and 9.2%, respectively. Independent predictors of in-hospital mortality were ejection fraction (EF) < 40% [adjusted Odds Ratio (aOR), 4.446, p < 0.001], culprit lesions in the left coronary artery (LCA) (aOR, 2.940, p < 0.001) compared with those in the right coronary artery, Killip class > II (aOR, 7.438; p < 0.001), chronic kidney disease (CKD) (aOR, 4.056; p < 0.001), final thrombolysis in myocardial infarction (TIMI) grades 0/1/2 (aOR, 1.809; p = 0.03), absence of robust collaterals (aOR, 17.309; p = 0.01) and hypertension (aOR, 0.449; p = 0.01).

**Conclusions:**

Among the consecutive patients with STEMI, the in-hospital mortality rate after p-PCI significantly improved in the second half. Not only CKD, Killip class > II, and EF < 40%, but also the angiographic findings such as culprit lesions in the LCA, absence of very robust collaterals, and final TIMI grades <3 were associated with an increased risk of in-hospital mortality.

## Introduction

Despite ongoing improvements in interventional technology, in-hospital mortality after the primary percutaneous coronary intervention (PCI) remains high among patients with some types of ST-elevation myocardial infarction (STEMI) [1–5].

Findings of several important studies have shown that the predictors of in-hospital mortality after STEMI include the Killip class, systolic blood pressure, heart rate, cardiac arrest, older age, prior heart failure, prior myocardial infarction (MI), peripheral arterial disease, chronic kidney disease (CKD), and elevated initial serum creatinine levels [1, 2]. Furthermore, other investigators have reported that other comorbidities related to atherosclerosis and blood test variables are associated with increased risks of in-hospital mortality in patients with STEMI [3–5].

However, data from consecutive STEMI populations have not been comprehensively evaluated because the data do not include detailed findings of angiography. Thus, few data describe relationships between in-hospital mortality and detailed findings from evaluations of the coronary artery in these patients after primary PCI. Furthermore, as most studies predate the primary PCI era, their findings may not apply to modern clinical practice. Therefore, this study aimed to assess angiographic variables associated with in-hospital mortality and to evaluate its impact on in-hospital mortality in consecutive patients with STEMI in the primary PCI era.

## Materials and methods

### Research ethics

Written informed consent was obtained from each patient or his/her relatives before or after PCI. This study was approved by Ogaki Municipal hospitals’ medical ethics committees, and it was conducted in accordance with the Declaration of Helsinki.

### Study design and population

Data from patients with STEMI who underwent primary PCI between January 2004 and December 2018 were extracted from the Ogaki Municipal Hospital’s database. The disease registry is an ongoing, retrospective, single-center hospital-based registry according to coronary heart disease. The catchment area of Ogaki Municipal Hospital is near Nagoya city and its outskirts in Seino area, western Gifu Prefecture; the total population of the area was about 372,000 in 2015. Patients were excluded if their onset-to-door time was > 24 h, their STEMI onset time was unclear, they had culprit lesions but did not undergo PCI, they underwent coronary artery bypass grafting (CABG) instead of PCI, they were treated with thrombolysis therapy or their culprit lesions were within bypass grafts. Furthermore, in patients who had multiple occurrences of STEMI, follow-up data was used for the first event of STEMI. The study period was divided into the first half and second half periods according to half the number of patients with STEMI who underwent PCI. Clinical data based on medical records on admission and angiographic data at index procedure were collected. Follow-up data were obtained from hospital charts.

#### Study endpoint

The study’s endpoint were 30-day mortality and in-hospital mortality. In addition, 30-day mortality and in-hospital mortality in each angiographic finding was evaluated.

#### Definitions

STEMI was defined as reported in previous studies [6, 7]. Left ventricular ejection fraction (EF) was measured during hospitalization, using the Teichholz method. EF was evaluated using the modified Simpson`s biplane method with two-dimensional apical, two-chamber, and four-chamber views in patients with local asynergy. EF <40% was defined as reduced EF as JCS guideline 2017 reported [8]. CKD was defined as an estimated glomerular filtration rate (eGFR) < 60 mL/min.1.73 m^2^ [9], which was calculated using a revised equation for Japanese people, as follows: eGFR (mL/min.1.73 m^2^) = 194 × serum creatinine ^−1.094^ × age ^−0.287^ × 0.739 (if female) [10]. Hypertension was defined as current or previous treatment with antihypertensive medication. Right dominant was defined when only the origin of the posterior descending artery (PDA) was from RCA, as previously reported [11]. The collateral circulation information was evaluated using Rentrop criteria. Rentrop grade III was defined as presence of very robust collaterals [12]. Initial arterial patency and restoration of arterial patency after PCI were evaluated according to TIMI (Thrombolysis in Myocardial Infarction) grade [13]. Chronic total occlusion (CTO) was defined as complete occlusion with TIMI 0 flow lasting at least 3 months regardless of the occluded location. Mechanical complications was defined as ventricular septal perforation (VSP), and left ventricular free wall rupture (LVFWR). Cardiovascular (CV) death includes death resulting from an acute myocardial infarction (MI), sudden cardiac death, death due to heart failure, death due to stroke, death due to CV procedures, death due to CV hemorrhage, and death due to other CV cause. Multiple factors due to STEMI within 30 days was classified into CV death [14]. Non-Cardiovascular death included as follows: Infection which was defined as any infection disease, and sepsis, gastrointestinal tract bleeding, suffocation, and pulmonary disease. In addition, multiple factors 30 days later after STEMI was classified into Non-Cardiovascular death.

### Statistical analyses

The continuous variables are expressed as the means and the standard deviations or as the medians and the interquartile ranges (IQRs) (Q1–Q3). The categorical covariates are expressed as numbers and percentages. Student’s t-test was used to analyze the continuous variables with normal distributions, and the Mann-Whitney U test was used to analyze the continuous variables with non-normal distributions. The categorical covariates were compared using the chi-square test. Logistic regression analysis with forced entry method was performed to identify independent predictors of in-hospital mortality after PCI for STEMI using variables that had values of p < 0.05 in the univariate analysis, age, and sex. The adjusted odds ratios (aOR) and 95% confidence intervals (CIs) were estimated. Goodness-of-fit was assessed using the Hosmer-Lemeshow test. IBM^®^SPSS^®^ software, version 26 (IBM Corporation, Armonk, NY, USA) was used to perform the statistical analyses. All of the p-values were 2-tailed, and a value of p < 0.05 was considered statistically significant.

## Results and discussion

### Patients’ characteristics

**Fig 1:**
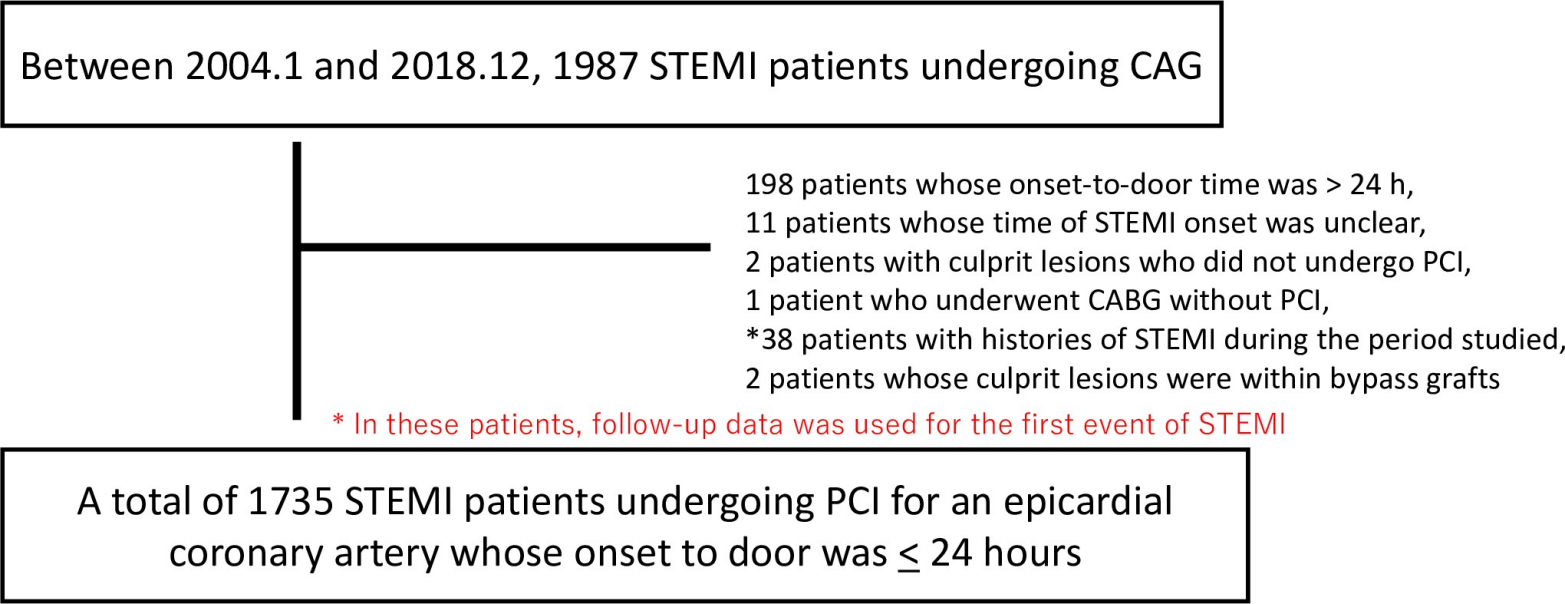
Study population all patients. STEMI, ST elevated myocardial infarction; CAG, coronary angiography; CABG, coronary artery bypass grafting.

During the period studied, 1,987 patients with STEMI underwent primary PCI. Of these, a total of 1,735 patients with STEMI were included in this study (Fig 1). Median Hospital days was 15.0 days (IQR: 12.0–21.0). Table 1 presents the patients’ baseline clinical and angiographic characteristics. The study cohort’s mean age was 68.5 years, and 24.6% of the patients were women. On admission, 7.7% of the patients met the criteria for Killip class IV. Diabetes mellitus and CKD were present in 34.0% and 39.1% of the patients, respectively. Right dominant coronary artery and CTO were present in 94.4% and 12.0% of the patients, respectively. During hospitalization, 4.9% and 29.3% of the patients required percutaneous cardiopulmonary support (PCPS) and intra-aortic balloon pumps (IABPs), respectively (Table 1). IABP were used for 4.0 days (IQR: 3.0–6.0) and PCPS were used for 3.0 days (IQR: 2.0–6.0).

**Table 1:** Baseline patient, angiographic, and procedural characteristics (n = 1735).

	Overall N = 1735	In hospital death (+) n = 159	In-hospital death (-) n = 1576	P value
**Patient clinical characteristics**				
Age, years	68.5 ± 12.2	74.6 ± 12.8	67.9 ± 12.0	<0.001
Over 80 years old, n (%)	353 (20.3)	64 (40.3)	289 (18.3)	<0.001
Female, n (%)	427 (24.6)	51 (32.1)	376 (23.9)	0.026
Body mass index < 22kg/m^2^, n (%)	565 (32.9)	79 (51.6)	486 (31.1)	<0.001
Current Smoker, n (%)	811 (47.7)	59 (40.4)	752 (48.4)	0.003
Prior MI, n (%)	177 (10.2)	22 (12.4)	155 (9.8)	0.129
Prior CABG, n (%)	12 (0.7)	1 (0.6)	11 (0.7)	1.000
Prior ischemic stroke, n (%)	147 (8.5)	24 (15.1)	123 (7.8)	<0.001
Dyslipidemia, n (%)	1320 (76.2)	114 (73.1)	1206 (76.5)	0.326
Diabetes mellitus, n (%)	589 (34.0)	53 (33.8)	536 (34.0)	1.000
Hypertension, n (%)	1437 (82.8)	116 (73.0)	1321 (83.8)	0.001
CKD, Estimated GFR < 60, n (%) *	678 (39.1)	122 (77.2)	556 (35.3)	<0.001
Peripheral artery disease	159 (9.2)	3 (5.0)	156 (9.3)	0.361
Ejection fraction < 40% **	182 (11.0)	48 (49.0)	134 (8.6)	<0.001
**Findings at presentation**				
CPA, n (%)	80 (4.6)	52 (32.7)	28 (1.8)	<0.001
Killip class > II, n (%)	429 (24.7)	121 (76.1)	308 (19.6)	<0.001
Killip class IV, n (%)	134 (7.7)	65 (40.9)	69 (4.4)	<0.001
Killip class, n (%)				
I, n (%)	1305 (75.3)	38 (23.9)	1267 (80.4)	<0,001
II, n (%)	215 (12.4)	32 (20.1)	183 (11.6)	
III, n (%)	80 (4.6)	24 (15.1)	56 (3.6)	
IV, n (%)	134 (7.7)	65 (40.9)	69 (4.4)	
**Angiographic characteristics**				
**Culprit vessel**				
LMT, n (%)	43 (2.5)	20 (12.5)	23 (1.3)	<0.001
LAD, n (%)	821 (47.3)	85 (53.5)	736 (46.7)	
LCx, n (%)	180 (10.4)	19 (11.9)	161 (10.2)	
RCA, n (%)	691 (39.8)	35 (22.0)	656 (41.6)	
**Initial TIMI grade**				
0	911 (52.5)	96 (60.4)	815 (51.7)	0.023
1	219 (12.6)	24 (15.1)	195 (12.4)	
2	442 (25.5)	25 (15.7)	417 (26.5)	
3	163 (9.4)	14 (8.6)	149 (9.5)	
Initial TIMI 0/1, n (%)	1130 (65.1)	120 (75.5)	1010 (64.1)	0.004
Final TIMI grade				
0, 1	33 (1.9)	10 (6.3)	23 (1.5)	<0.001
2	353 (20.3)	57 (35.8)	296 (18.8)	
3	1349 (77.8)	92 (57.9)	1257 (79.8)	
Final TIMI 3, n (%)	1349 (77.8)	92 (57.9)	1257 (79.8)	<0.001
Rentrop grade				
0	919 (53.0)	104 (65.4)	815 (51.7)	0.004
1	395 (22.8)	25 (15.7)	370 (23.5)	
2	274 (15.8)	24 (15.1)	250 (15.9)	
3	147 (8.5)	6 (3.8)	141 (8.9)	
Rentrop 3, n (%)	147 (8.5)	6 (3.8)	141 (8.9)	0.024
3-vessel disease, n (%)	215 (12.4)	32 (20.1)	183 (11.6)	0.003
Chronic total occlusion, n (%)	209 (12.0)	37 (23.3)	172 (10.9)	<0.001
Right dominant coronary artery	1638 (94.4)	154 (96.9)	1484 (94.2)	0.204
**Procedural characteristics**				
**PCI strategy**				
POBA	171 (9.9)	20 (12.6)	151 (9.6)	0.262
Stent	1564 (90.1)	139 (87.4)	1425 (90.2)	
**Type of stent (n = 1564)**				
BMS	951 (60.8)	95 (68.3)	856 (60.1)	0.144
1^st^ DES	23 (1.5)	1 (0.7)	22 (1.5)	
2^nd^ DES	590 (37.7)	43 (30.9)	547 (38.4)	
IABP	508 (29.3)	107 (67.3)	401 (25.4)	<0.001
PCPS before intervention	50 (2.9)	36 (22.6)	14 (0.9)	<0.001
PCPS during PCI	17 (1.0)	11 (6.9)	6 (0.4)	<0.001
**Others**				
Onset to door, hour	2.0 (1.0–5.0)	2.0 (1.0–5.0)	2.0 (1.0–4.3)	0.560
Onset to door < 12 hours	1562 (90.0)	139 (87.4)	1423 (90.3)	0.265
Door to balloon time, min	85.0 (65.0–120.0)	101.0 (64.0–118.3)	84.0 (64.0–118.3)	0.061
Peak CPK	2204.0 (1061.5–3930.3)	3833.0 (1633.0–8781.3)	2133.5 (1029.8–3684.8)	<0.001

Values are numbers (%) or mean ± SD. Values are also presented as median (Q1-Q3).

* Data was available in 1734 patients.

** Data was available in 1654 patients.

MI, myocardial infarction; PCI, percutaneous coronary intervention; CABG, coronary artery bypass grafting; CKD, chronic kidney disease; GFR, glomerular filtration rate; CPA, cardiopulmonary arrest; LMT, left main trunk; LAD, left anterior descending artery; LCx, left circumflex artery; RCA, right coronary artery; TIMI, Thrombolysis in myocardial infarction; CPK, creatine kinase; POBA, plain old balloon angioplasty; DES, drug eluting stent, IABP, intra-aortic balloon pumping; PCPS, Percutaneous cardiopulmonary support

### 30-day mortality and in-hospital outcomes

**Fig 2:**
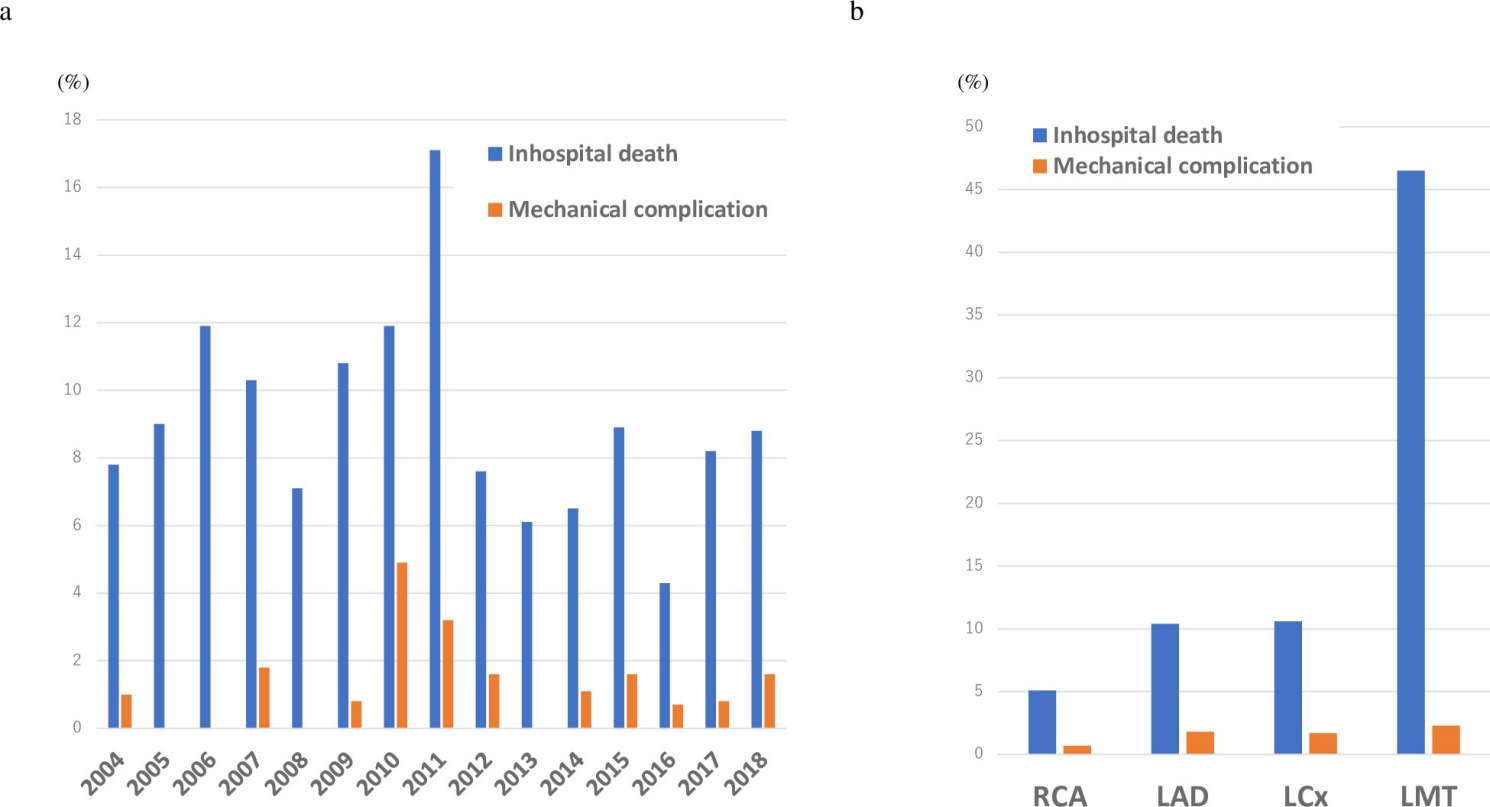
In-hospital outcome according to procedural year (a) and culprit vessels (b). RCA, right coronary artery; LAD, left anterior descending artery; LCx, left circumflex artery; LMT, left main trunk.

The 30-day mortality rate and in-hospital mortality rate were 7.7% and 9.2%, respectively. The trend of in-hospital mortality was illustrated in Fig 2A. The in-hospital mortality was 10.4% during the first half and 7.5% during the second half of the period studied (p = 0.02). Mechanical complications, which were associated with a high in-hospital mortality rate (70.8%), occurred in 1.4% of the patients. LVFWR occurred in 0.8% of the patients, and 0.3% and 0.5% of the patients had oozing and blowout type LVFWRs, respectively. VSP occurred in 0.6% of the patients (Table 2). The percentages of the causes of in-hospital death are shown in Table 3. The most frequent cause was cardiac shock (49.1%) followed by mechanical complications (9.4%) and fatal arrhythmia (6.9%).

**Table 2:** In-hospital clinical outcomes (n = 1735).

	Overall N = 1735	In hospital death (+) n = 159	In-hospital death (-) n = 1576	P value
**• In-hospital events**				
CABG	10 (0.5)	2 (1.3)	8 (0.5)	0.234
PCPS after PCI	18 (1.0)	12 (7.5)	6 (0.4)	<0.001
Acute stent thrombosis, n (%)	2 (0.1)	0 (0)	2 (0.1)	1.000
Sub-acute/late stent thrombosis, n (%)	10 (0.6)	2 (1.3)	8 (0.5)	0.234
Mechanical complications	24 (1.4)	17 (10.7)	7 (0.4)	<0.001
LVFWR (blow out)	8 (0.5)	7 (4.4)	1 (0.1)	
LVFWR (oozing)	6 (0.3)	4 (2.5)	2 (0.1)	
VSP	10 (0.6)	6 (3.8)	4 (0.3)	

Values are numbers (%)

CABG, coronary artery bypass grafting; PCPS, Percutaneous cardiopulmonary support; PCI, percutaneous coronary intervention; LVFWR, left ventricular free wall rupture; VSP, ventricular septum perforation

**Table 3:** Cause of in-hospital death (n = 159).

The reason of in-hospital death	N (%)
**• Cardiovascular death**	
Cardiac shock	78 (49.1)
Mechanical complications	15 (9.4)
Fatal arrythmia	11 (6.9)
Sudden cardiac death	3 (1.9)
Stent thrombosis	2 (1.3)
Multiple factors due to STEMI within 30 days	10 (6.3)
Aortic stenosis	2 (1.3)
Aortic aneurysm rupture	2 (1.3)
Ascending Aortic dissection	3 (1.9)
Death because of cardiovascular surgical Procedures	2 (1.3)
Ischemic stroke	5 (3.1)
hypoxic encephalopathy	1 (0.6)
Intracranial bleeding	1 (0.6)
Pulmonal embolism	1 (0.6)
**• Non-Cardiovascular death**	
Infection	9 (5.7)
Renal failure	3 (1.9)
Multiple factors	7 (4.4)
Gastrointestinal tract Bleeding	1 (0.6)
Suffocation	2 (1.3)
Pulmonary disease	1 (0.6)

Values are numbers (%)

### Predictors of in-hospital mortality

Compared with the patients who survived STEMI, those who died during hospitalization were more likely to be older, women, have BMIs < 22 kg/m^2^, have histories of ischemic stroke, hypertension, and CKD, be categorized as Killip class IV on admission, require PCPS and IABPs, and have the 3-vessel disease (TVD). The in-hospital mortality rates were significantly different according to the culprit lesion. It was higher in the patients whose culprit lesions were in the left main stem (LMS), followed by the left circumflex artery (LCx), left anterior descending artery (LAD), and right coronary artery (RCA). Odds ratio for LAD, LCx, LM compared with RCA as a culprit were 2.165 (p < 0.001), 2.212 (p = 0.008), and 16.298 (p < 0.001) (Fig 2B).

The patients who died in hospital were more likely to have lower initial and final thrombolysis in myocardial infarction (TIMI) grades and undeveloped robust collaterals compared with the patients who survived STEMI (Table 1).

Independent predictors of in-hospital mortality were ejection fraction (EF) < 40% [adjusted Odds Ratio (aOR), 4.446, p < 0.001], culprit lesions in the left coronary artery (LCA) (aOR, 2.940, p < 0.001) compared with those in the right coronary artery, Killip class > II (aOR, 7.438; p < 0.001), CKD (aOR, 4.056; p < 0.001), final thrombolysis in myocardial infarction (TIMI) grades 0/1/2 (aOR, 1.809; p = 0.03), without very robust collaterals (aOR, 17.309; p = 0.01) and hypertension (aOR, 0.449; p = 0.01) (Table 4).

**Table 4:** Univariate and multivariate regression analysis for the association between in-hospital mortality and clinical findings.

	Univariate analysis			Multivariate analysis		
	All-cause mortality					
Factors for predicting	OR	95% CI	p value	Adjusted OR	95% CI	p value
EF <40%	10.187	6.602–15.721	0.001<	4.446	2.613–7.566	0.001<
Killip I vs. II/III/IV	13.099	8.912–19.253	0.001<	7.438	4.323–12.796	0.001<
Estimated GFR < 60	6.217	4.227–9.144	0.001<	4.056	2.260–7.279	0.001<
RCA vs. LAD/LCx/LM	2.526	1.713–3.725	0.001<	2.940	1.594–5.422	0.001
Rentrop 0.1.2	2.506	1.088–5.768	0.031	17.309	2.030–147.585	0.009
Hypertension	0.521	0.358–0.757	0.001	0.449	0.247–0.817	0.009
Final TIMI 0.1.2	2.870	2.047–4.023	0.001<	1.809	1.068–3.065	0.027
Initial TIMI 0.1	1.724	1.184–2.510	0.004	1.652	0.952–2.868	0.075
age	1.052	1.036–1.068	0.001<	1.018	0.993–1.044	0.152
3-vessel disease	1.918	1.264–2.911	0.002	1.766	0.861–3.621	0.121
BMI (per 1 kg/m^2^)	0.904	0.861–0.948	0.001<	0.980	0.916–1.049	0.565
Prior ischemic stroke	2.129	1.345–3.369	0.001	1.158	0.551–2.435	0.699
CTO	2.476	1.659–3.695	0.001<	1.820	0.851–3.893	0.123
Female	1.507	1.059–2.144	0.023	1.142	0.647–2.018	0.647

Abbreviations as in Tables 1 and 2.

OR, Odds ratio; CI, confidence interval; BMI, Body mass index; CTO, Chronic total occlusion

In this study, independent predictors of in-hospital mortality were evaluated in consecutive patients with STEMI who underwent PCI in the modern primary PCI era. This study’s key findings showed that 9.2% of the patients with STEMI died during hospitalization. Second, mechanical complications occurred in a total of 1.4%, leading to extremely high in-hospital mortality. In addition, a certain number of STEMI patients whose hemodynamics was unstable resulted in high in-hospital mortality. Third, this study showed that EF < 40%, Killip class >II, CKD, vessel containing the culprit lesion, insufficient robust collaterals, final TIMI grade < 3 were associated with increased risks of in-hospital mortality.

In general, the in-hospital mortality after primary PCI for STEMI ranges from 2.5% to 9.4% in Japan [15–19] from 2.2% to 7.9% among unselected patients with STEMI in the national registries of the European Society of Cardiology’s member countries [20, 21], from 4.6% to 6.3% in several registries in the United States [5, 22]. Our data showed that the in-hospital mortality rate for consecutive patients with STEMI was 9.2% in overall, which is comparable with previously reported rates [15–18, 20, 21]. In patients with STEMI, primary PCI and early recanalization of occluded arteries can dramatically improve in-hospital mortality [23]. However, contrary to expectations, advances in interventional devices and promotion of early reperfusion have not continued to reduce the in-hospital mortality [24]. In correspondence with the previous report,(24) this study showed that the rate of in-hospital mortality still remained high.

There are several potential explanations for this remaining issue. First, there are yearly around 10% of STEMI patients who present with cardiac shock [25]. Similarly, our data included 7.7% of the patients with Killip class IV, 4.6% of those who presented with cardiopulmonary arrest, and 4.9% of those who needed PCPS support. Therefore, hemodynamic instability, such as higher Killip classification, undoubtedly caused high mortality rates, even in the primary PCI era. Second, mechanical complications, which are associated with extremely high in-hospital mortality rates, continue to occur in around 1% of patients, even after the era of primary PCI [26, 27]. This study’s findings showed that mechanical complications occurred in 1.5% of the patients and that 0.8% and 0.6% of the patients had LVFWR and VSP, respectively, which concurs with previous studies’ findings [6, 28]. Mechanical complications have persisted as one of the most important causes of in-hospital death [29]. Hence, we might consider the indications for IABPs, which can reduce the afterload, and the early administration of β-blockers and angiotensin-converting enzyme inhibitors in order to prevent transitory hypertension with exercise in elderly women or in those with a delayed first MI who are at a high risk of mechanical complications [6, 28, 30, 31]. Finally, although our institutes are regional core hospitals that cover large areas, preventive approaches have not reduced pre-hospital delays or improved access to early reperfusion. In addition, the median door to balloon time was 85 minutes and relatively longer in this study compared to that in the Guidelines [7]. This delay in reperfusion might lead to worse clinical outcomes in some patients after p-PCI. As the cardiogenic shock is exacerbated by delays in recanalization [25], we must create an emergency STEMI system and raise awareness about coronary artery disease throughout the community to shorten both door to balloon time and onset to reperfusion time.

On the other hand, all patients with STEMI should undergo early assessments of their short-term risk. Previously, several clinical markers indicated a high risk of in-hospital mortality [1, 2, 4, 5]. However, most of these studies predate the primary PCI era, and their results may not be applicable to modern clinical practice. Because some of the risk assessments lacked angiographic variables, our analysis highlights the synergistic and prognostic impacts of both the clinical and angiographic variables on clinical outcomes. First, our data suggested that when there were very robust coronary collaterals, in-hospital mortality might be low in patients with STEMI because robust collaterals to infarct-related arteries could stabilize the hemodynamics, which is in line with previous results [32, 33]. Furthermore, robust collaterals could provide myocardial protective effects, such as improved functional recovery and infarct size reduction in patients with STEMI, as an MRI study showed [34]. Second, it is important to recognize that the optimal achievement of final TIMI grades 3 might reduce the risk of in-hospital mortality during p-PCI. In general, suboptimal flow and final TIMI grades 0/1/2 after a primary PCI are well known to be caused by microvascular dysfunction via vascular constriction, distal microembolization, and endothelial dysfunction, which are secondary to endothelial injury, plugging by platelets, neutrophils, erythrocytes, and intracellular and interstitial edema [35, 36]. Therefore, our findings remind us of the importance of achieving a final TIMI grade of 3 as part of the successful treatment of patients with STEMI. Third, our analysis recognizes the novelty that the left coronary artery, including not only LM, LAD, but also LCx as a culprit lesion, was associated with an increased risk of in-hospital mortality compared with RCA. In other words, RCA as a culprit lesion might be benign compared with LCA, which is in line with previous reports. Generally, LMS and proximal LAD are well-known predictors of mortality in STEMI patients due to the large territory of the myocardium. However, the report regarding the impact of LCx is limited [37]. Therefore, this study firstly implies that we reconsider the impact of LCx as a culprit lesion in patients with STEMI because the acute occlusion of LCx might have more impact than we had expected. We speculated that delayed diagnoses based on electrocardiograms [38, 39] and the impact of left dominancy, a dominant LCX artery which results in a high proportion of patients with cardiogenic shock [40], might influence in-hospital mortality. However, the precise mechanism has still remained unclear. Hence, further studies investigating the impact of the LCx on clinical outcomes are needed to clarify the relationships between culprit lesions in the LCx and in-hospital mortality.

### Study limitations

This study has several limitations. First, this was a non-randomized, retrospective study with a relatively small number. Second, we did not perform any comparison between STEMI patients who were not treated with primary PCI and those who were treated conservatively. Third, this study’s findings showed that a history of hypertension was a benign predictor of in-hospital mortality due to survival bias and ambiguity driven by a lack of data on medication before admission, which is inconsistent with previous reports [41, 42]. Therefore, hypertension must be carefully scrutinized as a predictor of in-hospital mortality. Fourth, we did not include the systolic blood pressure or the heart rate on admission, or cardiopulmonary arrest in the final logistic regression models due to the presence of multicollinearity. Fifth, our study lacked the data of cardiac valvular disease. Sixth, we did not analyze the impact of gender on in-hospital mortality because of limited number. Further studies are necessary to show the impact of gender difference on in-hospital mortality after STEMI. Seventh, this study showed the improvement of in-hospital mortality in the second half compared to the first half. Because this finding is important, we would discuss this issue in the next paper. Finally, our study lacked an independent systematic quantitative coronary analysis, and it did not include data based on quantitative coronary angiography [43]. To clarify the independent predictors associated with in-hospital mortality, a larger sample of patients who have undergone PCI for STEMI and dedicated angiographic data are required.

## Conclusions

Among the consecutive patients with STEMI, the in-hospital mortality rate after p-PCI was significantly improved in the second half of the period studied. Not only CKD, Killip class > II, and EF < 40%, but also the angiographic findings such as culprit lesions in the LCA, absence of very robust collaterals, and final TIMI grades <3 were associated with an increased risk of in-hospital mortality.

## Supporting information

S1 Checklist The RECORD statement–checklist of items, extended from the STROBE statement, that should be reported in observational studies using routinely collected health data. (DOCX)
